# Association of Peripheral TNF-α and IFN-γ Expression With Serum Uric Acid Levels and Clinical Disease Severity in Patients With Parkinson’s Disease: A Cross-Sectional Study

**DOI:** 10.7759/cureus.111772

**Published:** 2026-06-29

**Authors:** Joby Wilson, Seetha Rami Reddy Mallampati, Burhanuddin Parawala, Varunkrishnan G, Arun Raja

**Affiliations:** 1 Internal Medicine, Vinayaka Mission's Medical College and Hospital, Vinayaka Mission’s Research Foundation (Deemed to be University), Karaikal, IND; 2 Internal Medicine, Vinayaka Mission's Medical College and Hospital, Vinayaka Mission's Research Foundation (Deemed to be University), Karaikal, IND; 3 General Medicine, Vinayaka Mission's Medical College and Hospital, Vinayaka Mission’s Research Foundation (Deemed to be University), Karaikal, IND; 4 Preventive Medicine, Vinayaka Mission's Medical College and Hospital, Vinayaka Mission’s Research Foundation (Deemed to be University), Karaikal, IND

**Keywords:** biomarkers, ifn-γ, neuroinflammation, oxidative stress, parkinson’s disease, qrt-pcr, tnf-α, uric acid

## Abstract

Background

Parkinson’s disease (PD) is a progressive neurodegenerative disorder characterized by dopaminergic neuronal loss, neuroinflammation, and oxidative stress. Pro-inflammatory cytokines such as TNF-α and IFN-γ contribute to neuronal injury, whereas uric acid acts as an important endogenous antioxidant. The interaction between inflammatory cytokines and antioxidant status in PD remains incompletely understood.

Methods

This hospital-based cross-sectional observational study included 44 patients with clinically diagnosed PD. Disease severity was assessed using the Webster Rating Scale (WRS) and the Hoehn and Yahr (H&Y) staging system. Serum uric acid levels were estimated using the uricase-peroxidase method. Peripheral blood TNF-α and IFN-γ gene expression levels were quantified using quantitative real-time polymerase chain reaction (qRT-PCR) and analyzed using the 2^-ΔΔCt method. Correlation analysis and logistic regression were performed to evaluate associations between biomarkers and disease severity.

Results

The mean age of participants was 73.16 ± 8.80 years, with a male predominance of 29 (65.9%). TNF-α and IFN-γ expression levels progressively increased with advancing disease severity among patients with PD. Both cytokines demonstrated significant positive correlations with WRS and H&Y scores. In contrast, serum uric acid levels progressively declined with increasing disease severity and showed significant inverse correlations with TNF-α and IFN-γ expression. On multivariate logistic regression analysis, elevated TNF-α and IFN-γ expression remained independently associated with advanced disease severity, whereas higher serum uric acid levels demonstrated a protective association.

Conclusion

Higher TNF-α and IFN-γ expression, along with lower serum uric acid levels, were associated with greater disease severity. These findings suggest their potential utility as candidate biomarkers, which require validation in larger longitudinal studies.

## Introduction

Parkinson's disease (PD) is a progressive neurodegenerative disorder marked by the selective loss of dopaminergic neurons in the substantia nigra pars compacta, resulting in striatal dopamine deficiency and impaired motor function. Clinically, PD manifests with resting tremor, bradykinesia, rigidity, and postural instability as its hallmark motor features, while non-motor manifestations, including depression, autonomic disturbances, sleep dysfunction, and cognitive decline, substantially add to the overall disease burden and diminished quality of life. Despite decades of research, the precise mechanisms underlying PD pathogenesis remain elusive and are thought to arise from a multifactorial interplay involving genetic predisposition, mitochondrial impairment, abnormal protein aggregation, oxidative injury, and neuroinflammatory processes [1].

Growing evidence highlights chronic neuroinflammation as a pivotal driver of both the onset and advancement of PD. Microglial activation results in the sustained release of pro-inflammatory mediators and neurotoxic substances that inflict injury upon dopaminergic neurons. Among these, TNF-α and IFN-γ have emerged as key cytokines implicated in neuronal degeneration, operating through apoptotic pathway activation, mitochondrial compromise, nitric oxide overproduction, and oxidative tissue damage. Detectable elevations of TNF-α and IFN-γ in both serum and cerebrospinal fluid of PD patients point to the existence of ongoing systemic as well as central nervous system inflammatory activity [2,3].

Oxidative stress represents an additional and closely interrelated pathogenic mechanism in PD. An imbalance between reactive oxygen species generation and antioxidant capacity results in lipid peroxidation, mitochondrial structural damage, and eventual neuronal cell death. Uric acid, among the most prevalent endogenous antioxidants in the human body, confers neuroprotection via scavenging of free radicals and chelation of redox-active transition metals. Epidemiological evidence has consistently linked lower circulating uric acid concentrations to a heightened risk and more rapid progression of PD [4,5]. Nevertheless, studies concurrently examining both inflammatory cytokine profiles and antioxidant capacity in the context of clinical disease severity remain scarce. Both TNF-α and IFN-γ were selected because they are among the most extensively studied pro-inflammatory cytokines implicated in microglial activation, neuronal injury, and disease progression in PD. Uric acid was selected as a representative endogenous antioxidant due to its established neuroprotective role and reported association with disease risk and progression. While previous studies have evaluated individual inflammatory markers or serum uric acid separately, limited evidence exists regarding the simultaneous assessment of these inflammatory and antioxidant biomarkers in relation to clinical disease severity. Addressing this gap may provide a more comprehensive understanding of the interplay between neuroinflammation, oxidative stress, and disease progression in PD. The present study was therefore undertaken to assess the relationship between peripheral TNF-α and IFN-γ expression, serum uric acid levels, and clinical severity in patients with PD.

## Materials and methods

This hospital-based cross-sectional study was carried out over 18 months in the Department of General Medicine at Vinayaka Mission's Medical College and Hospital, Karaikal, Tamil Nadu, India. Ethical clearance was obtained from the Institutional Ethics Committee prior to commencement of the study (approval no. VMMC/GENMED/2022/JULY/07). The study was conducted in conformity with the ethical standards of the Declaration of Helsinki (2013), and written informed consent was secured from every participant before enrollment.

A total of 44 patients with a clinical diagnosis of Parkinson's disease (PD), established using the United Kingdom Parkinson's Disease Society Brain Bank diagnostic criteria, were enrolled. Inclusion required a confirmed diagnosis of idiopathic PD, age 18 years or above, and willingness to participate. Patients were excluded if they had secondary Parkinsonism, autoimmune or chronic inflammatory conditions, malignancy, cerebrovascular disease, Alzheimer's disease, other neurodegenerative disorders, or critical illness, or if they were pregnant or receiving immunosuppressive agents, corticosteroids, monoclonal antibodies, or biological therapies, so as to limit confounding factors. As this was an exploratory hospital-based study, the sample size was determined by the availability of eligible patients during the study period rather than a formal a priori sample size calculation. Baseline demographic and clinical data, including age, sex, illness duration, comorbidities, and medication history, were captured using a structured proforma, and all participants underwent thorough physical and neurological evaluation. Disease severity was graded using the Webster Rating Scale (WRS) [6], with scores categorized as mild (1-10), moderate (11-20), and severe (21-30), and independently staged using the Hoehn and Yahr (H&Y) system from stage I to stage V [7].

Peripheral venous blood (10 mL total) was drawn from each participant under aseptic conditions. Three milliliters were collected into a plain vacutainer for serum uric acid estimation, and the remaining 7 mL into an ethylenediaminetetraacetic acid (EDTA) vacutainer for cytokine gene expression analysis. Samples were centrifuged at 3000 rpm for 10 minutes at 4°C, after which serum aliquots were separated and stored at −80°C pending biochemical assay. Total RNA isolation from peripheral whole blood was performed using the TRIzol reagent method under RNase-free conditions. Briefly, 200 µL of whole blood was homogenized in 1 mL of TRIzol reagent, followed by chloroform phase separation, isopropanol precipitation, 70% ethanol washing, air drying, and resuspension in RNase-free water. The RNA yield and purity were assessed spectrophotometrically using a NanoDrop 2000 (Thermo Fisher Scientific, Waltham, MA, USA); samples with an A260/A280 ratio of 1.8-2.0 were accepted for downstream processing, and the RNA was stored at −80°C until use.

Complementary DNA (cDNA) synthesis was performed using the High-Capacity cDNA Reverse Transcription Kit (Applied Biosystems, Carlsbad, CA, USA) per the manufacturer's protocol in a 20 µL reaction containing an RNA template, reverse transcription buffer, dNTP mix, random primers, MultiScribe™ Reverse Transcriptase (Thermo Fisher Scientific), and nuclease-free water. Thermal cycling comprised primer annealing at 25°C, reverse transcription at 37°C, and enzyme inactivation at 85°C. Synthesised cDNA was stored at −20°C. Quantitative real-time PCR (qRT-PCR) for TNF-α and IFN-γ was performed on the Applied Biosystems 7500 platform using SYBR Green PCR Master Mix (Thermo Fisher Scientific), with gene-specific primers and GAPDH as the endogenous housekeeping reference. Reactions were run in triplicate in a 20 µL final volume, incorporating an initial denaturation step, 40 amplification cycles, and melt-curve analysis to verify amplification specificity. No-template controls were included in every run to detect potential contamination. Relative expression levels were normalized to GAPDH and quantified using the 2^-ΔΔCt method. All reactions were performed in triplicate, and the melt-curve analysis demonstrated single specific amplification products. No-template controls were included in each run to exclude contamination. Amplification efficiencies for all primer sets were within acceptable ranges for relative quantification.

Statistical analyses were conducted using SPSS Statistics version 25.0 (IBM Corp., Armonk, NY, USA). Continuous data are presented as mean ± standard deviation, and categorical data as frequencies with percentages. The distribution of continuous variables was assessed using the Shapiro-Wilk test and visual inspection of histograms. Variables meeting normality assumptions were analyzed using parametric methods. Associations between TNF-α and IFN-γ expression, serum uric acid levels, and disease severity scores were examined using Pearson's correlation coefficient. Bivariate logistic regression was performed to derive crude odds ratios (cORs) with 95% confidence intervals (CIs) for each variable in relation to advanced disease severity; variables meeting a p-value threshold of <0.20 were carried forward into a multivariate logistic regression model to compute adjusted odds ratios (aORs) with 95% CIs. As the analyses were exploratory and focused on a limited number of pre-specified biomarkers, adjustment for multiple comparisons was not performed. Therefore, the findings should be interpreted with appropriate caution. Statistical significance was defined as p <0.05.

## Results

A total of 44 patients with clinically diagnosed PD were included in the study. The mean age of the participants was 73.16 ± 8.80 years, with the majority belonging to the 71 to 80 years age group (15, 34.1%), followed by 61 to 70 years (13, 29.5%). Male participants constituted 65.9% of the study population, resulting in a male-to-female ratio of approximately 2:1. The mean duration of illness was 6.3 ± 3.5 years. Based on the WRS, 10 (22.7%) patients had early disease, 29 (65.9%) had moderate disease, and 5 (11.4%) had severe or advanced disease. According to the H&Y staging system, 17 (38.6%) participants were classified under stage III disease. Resting tremor was the most common motor manifestation, and with respect to comorbidities, hypertension was the most common comorbidity observed among the study participants (Table 1).

**Table 1:** Baseline clinical characteristics of the study participants WRS: Webster Rating Scale, H&Y: Hoehn and Yahr

Variables		N (%)/Mean ± SD
Age group (years)	< 60	5 (11.4)
	61-70	13 (29.5)
	71-80	15 (34.1)
	81-90	11 (25.0)
	Mean age (years)	73.16 ± 8.80
Gender	Male	29 (65.9)
	Female	15 (34.1)
	Disease duration (years)	6.3 ± 3.5
Disease severity (WRS)	Early illness	10 (22.7)
	Moderate	29 (65.9)
	Severe / Advanced	5 (11.4)
H&Y staging	Stage II	12 (27.3)
	Stage III	17 (38.6)
	Stage IV	12 (27.3)
	Stage V	3 (6.8)
Motor symptoms	Resting tremor	38 (86.4)
	Rigidity	35 (79.5)
	Bradykinesia	32 (72.7)
Comorbidities	Hypertension	21 (47.7)
	Type 2 diabetes mellitus	18 (40.9)

Analysis of inflammatory and oxidative stress biomarkers demonstrated a mean TNF-α relative fold expression of 2.82 ± 0.64 and a mean IFN-γ relative fold expression of 2.46 ± 0.59. The mean serum uric acid level was 4.12 ± 1.01 mg/dL. The mean serum albumin and hemoglobin levels were 3.62 ± 0.48 g/dL and 11.9 ± 1.8 g/dL, respectively (Table 2).

**Table 2:** Inflammatory and oxidative stress biomarkers in study participants *TNF-α and IFN-γ values represent relative fold expression calculated using the 2^-ΔΔCt method normalized to GAPDH.

Variable*	Mean ± SD	Median (IQR)
TNF-α (relative fold expression)	2.82 ± 0.64	2.74 (2.21–3.28)
IFN-γ (relative fold expression)	2.46 ± 0.59	2.38 (2.02–2.91)
Uric acid (mg/dL)	4.12 ± 1.01	4.03 (3.42–4.85)
Albumin (g/dL)	3.62 ± 0.48	3.65 (3.20–4.00)
Haemoglobin (g/dL)	11.9 ± 1.8	11.8 (10.6–13.0)

A progressive increase in inflammatory cytokine expression was observed with increasing disease severity. Based on WRS classification, the mean TNF-α expression increased from 1.96 ± 0.48 in patients with early disease to 3.12 ± 0.35 among patients with severe disease. Similarly, IFN-γ expression increased from 2.02 ± 0.37 in early disease to 3.01 ± 0.46 in severe disease. Both associations were statistically significant (p = 0.004 and p = 0.006, respectively). A similar trend was observed across advancing H&Y stages, where TNF-α expression increased from 1.49 ± 0.54 in stage II disease to 3.18 ± 0.72 in stage V disease, while IFN-γ expression increased from 2.03 ± 0.48 to 3.25 ± 0.70 across the same stages (p = 0.008 and p = 0.011, respectively) (Table 3).

**Table 3:** Association of inflammatory cytokine expression with disease severity scales WRS: Webster Rating Scale, H&Y: Hoehn and Yahr

Disease severity scale		TNF-α	IFN-γ	p-value
WRS classification	Early illness	1.96 ± 0.48	2.02 ± 0.37	
	Moderate	2.43 ± 0.44	2.41 ± 0.49	
	Severe/advanced	3.12 ± 0.35	3.01 ± 0.46	0.004 / 0.006
H&Y staging	Stage II	1.49 ± 0.54	2.03 ± 0.48	
	Stage III	2.02 ± 0.61	2.38 ± 0.56	
	Stage IV	2.61 ± 0.58	2.82 ± 0.64	
	Stage V	3.18 ± 0.72	3.25 ± 0.70	0.008 / 0.011

Correlation analysis demonstrated significant positive associations between inflammatory cytokine expression and disease severity scores. The TNF-α expression showed positive correlations with WRS (r = 0.49, p = 0.002) and H&Y staging scores (r = 0.45, p = 0.005). Similarly, IFN-γ expression demonstrated positive correlations with WRS (r = 0.46, p = 0.004) and H&Y staging scores (r = 0.42, p = 0.008). In contrast, serum uric acid levels demonstrated strong inverse correlations with both WRS scores (r = −0.74, p <0.001) and H&Y staging scores (r = −0.73, p <0.001) (Table 4).

**Table 4:** Correlation of disease severity scores with inflammatory cytokines and serum uric acid WRS: Webster Rating Scale, H&Y: Hoehn and Yahr

Disease severity scale		IFN-γ	TNF-α	Uric acid
WRS	r-value	0.46	0.49	-0.74
	p-value	0.004	0.002	<0.001
H&Y scale	r-value	0.42	0.45	-0.73
	p-value	0.008	0.005	<0.001

**Figure 1:**
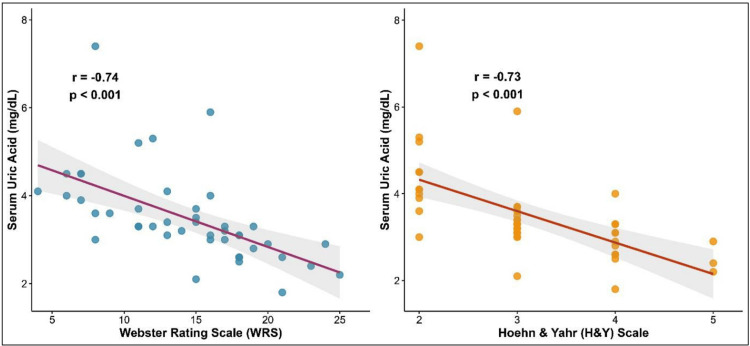
Correlation of serum uric acid levels with disease severity in PD Scatter plots showing the relationship between serum uric acid levels and WRS scores (left panel) and the H&Y staging (right panel) among 44 patients with PD. Solid lines represent fitted linear regression lines, and shaded areas indicate the 95% CI around the regression estimates. All observations were included in the analysis. PD: Parkinson’s disease, WRS: Webster Rating Scale, H&Y: Hoehn & Yahr

Serum uric acid levels progressively declined with increasing disease severity. Patients with advanced disease demonstrated lower serum uric acid concentrations compared to those with early-stage disease. Scatter plot analysis further demonstrated significant inverse relationships between serum uric acid levels and disease severity assessed using both the WRS and the H&Y staging system (Figure 1). Further analysis demonstrated significant negative correlations between serum uric acid and inflammatory cytokine expression. Serum uric acid negatively correlated with TNF-α expression (r = −0.41, p = 0.004) and IFN-γ expression (r = −0.39, p = 0.006). A mild positive correlation was observed between serum albumin and uric acid levels (r = 0.32, p = 0.03). However, hemoglobin levels and comorbid conditions such as hypertension and diabetes mellitus did not demonstrate statistically significant associations with inflammatory cytokine expression or serum uric acid levels.

Bivariate logistic regression analysis demonstrated that higher TNF-α expression (cOR: 2.48, 95% CI: 1.39-4.42; p = 0.002), increased IFN-γ expression (cOR: 2.11, 95% CI: 1.23-3.64; p = 0.006), lower serum uric acid levels (cOR: 0.52, 95% CI: 0.33-0.82; p = 0.004), and longer disease duration (cOR: 1.34, 95% CI: 1.06-1.69; p = 0.01) were significantly associated with advanced disease severity. After adjustment for age, sex, and disease duration in multivariate logistic regression analysis, TNF-α expression (aOR: 2.14, 95% CI: 1.18-3.87; p = 0.01) and IFN-γ expression (aOR: 1.92, 95% CI: 1.07-3.41; p = 0.02) remained independently associated with greater disease severity. In contrast, higher serum uric acid levels demonstrated an inverse association with advanced disease severity (aOR: 0.58, 95% CI: 0.36-0.91; p = 0.01). Disease duration also retained a modest independent association with increasing disease severity (aOR: 1.29, 95% CI: 1.01-1.66; p = 0.04).

## Discussion

The present study examined the interplay between peripheral inflammatory cytokine expression, oxidative stress markers, and clinical disease severity in patients with PD. Peripheral blood TNF-α and IFN-γ expression were found to be markedly elevated, while serum uric acid levels were concurrently reduced. Both cytokines exhibited significant positive associations with disease severity as graded by the WRS and H&Y staging, whereas serum uric acid showed a strong inverse relationship with severity scores. An additional inverse correlation between serum uric acid and inflammatory cytokine levels was noted, pointing towards a mechanistic link between impaired antioxidant capacity and heightened neuroinflammatory activity. On multivariate analysis, TNF-α and IFN-γ expression independently predicted advanced disease severity, while higher uric acid concentrations were associated with a protective effect. Taken together, these observations support an association between neuroinflammation, oxidative stress, and clinical disease severity in PD, although the cross-sectional nature of the study precludes conclusions regarding causality or underlying mechanisms.

Comparison with existing literature

The present findings resonate with accumulating evidence positioning neuroinflammation as an important feature associated with PD pathophysiology. The elevation in TNF-α expression noted in this study is in keeping with the seminal work by Mogi et al. that reported increased TNF-α concentrations in the serum and cerebrospinal fluid of PD patients, indicative of widespread systemic inflammatory activation [8]. Complementing this, Hunot et al. documented prominent microglial activation alongside upregulated TNF-α expression in the substantia nigra of PD brains, underscoring the contribution of sustained neuroinflammation to dopaminergic cell loss [9]. Further mechanistic insight comes from experimental data demonstrating that TNF-α drives neuronal apoptosis via TNFR1-dependent mitochondrial perturbation and caspase pathway engagement, thereby hastening the neurodegenerative process [10].

The stepwise increase in IFN-γ expression observed with worsening disease severity in the present study adds to prior evidence implicating adaptive immune activation in PD pathogenesis. Mount et al. established that IFN-γ potentiates microglial reactivity and directly mediates dopaminergic neuronal loss in experimental PD models [11]. In a parallel vein, Brochard et al. documented CD4+ and CD8+ T lymphocyte infiltration into the substantia nigra of affected individuals, underscoring the pathological significance of IFN-γ-driven cellular immune responses in disease advancement [12]. The positive correlations between IFN-γ expression and both WRS and H&Y scores identified here thus further substantiate the role of persistent immune-mediated signaling in the progression of motor disability in PD [13].

A further notable observation in this study was the progressive decline in serum uric acid with advancing disease severity. This finding is consistent with prior epidemiological and clinical data reporting reduced circulating urate in PD patients relative to healthy counterparts. De Lau et al. identified an association between lower serum uric acid and increased susceptibility to developing PD [14], while Ascherio et al. demonstrated that higher baseline urate concentrations predicted slower functional decline and deferred disability onset [15,16]. Experimental studies additionally corroborate a neuroprotective function of uric acid, attributed to its capacity to neutralize reactive oxygen species, chelate redox-active transition metals, and attenuate mitochondrial oxidative damage [17]. The inverse association between uric acid levels and disease severity observed in the present work, therefore, reinforces the view that compromised antioxidant defense is a meaningful contributor to PD progression.

Perhaps the most compelling observation in the present study was the significant inverse correlation between serum uric acid and inflammatory cytokine expression, suggesting that oxidative stress and neuroinflammation do not operate as independent processes but rather as interlinked and mutually amplifying mechanisms. This is consistent with experimental findings by Block et al., who showed that activated microglia drive simultaneous overproduction of reactive oxygen species and pro-inflammatory cytokines, perpetuating a self-sustaining cycle of neuronal injury [18]. Corroborating this, oxidative stress has independently been shown to upregulate inflammatory signaling through augmented cytokine release and nitric oxide synthesis [19]. The present data extend these experimental observations to a clinical setting, demonstrating that diminished antioxidant capacity is accompanied by significantly elevated inflammatory cytokine expression in patients with established PD. Notably, the inverse correlations between serum uric acid levels and both WRS (r = −0.74) and H&Y scores (r = −0.73) represent strong effect sizes, suggesting that antioxidant status may be closely linked to clinical disease severity. These findings support the potential relevance of oxidative stress pathways in disease progression and highlight the need for further longitudinal studies to determine whether serum uric acid may have prognostic utility in PD.

Clinical implications

The present findings highlight the potential relevance of inflammatory and oxidative stress biomarkers in understanding disease severity in PD. The observed association of elevated TNF-α and IFN-γ expression together with lower serum uric acid levels suggests that these markers may reflect underlying biological processes linked to disease progression. The concurrent assessment of inflammatory and antioxidant pathways may therefore provide complementary insights into the relationship between systemic inflammation, oxidative stress, and clinical severity in PD. Furthermore, the observed associations support continued investigation of therapeutic strategies targeting inflammatory and oxidative pathways. However, given the cross-sectional design and relatively small sample size, the clinical utility of these biomarkers and their potential role in guiding patient management require confirmation in larger prospective and longitudinal studies.

Strengths and limitations

A major strength of this study is the simultaneous evaluation of peripheral inflammatory cytokine expression, antioxidant status, and clinically validated disease severity measures, providing a comprehensive assessment of the association between systemic inflammation, oxidative stress, and disease severity in PD. The use of qRT-PCR for cytokine quantification and multivariable regression analysis further enhances the methodological rigor of the study. However, several limitations should be acknowledged. The relatively small sample size and cross-sectional design limit statistical power, preclude causal inference, and may affect the generalisability of the findings. A particularly important limitation is the absence of a healthy control group, which precluded direct comparison of cytokine expression and serum uric acid levels between patients with PD and unaffected individuals. Consequently, the findings should be interpreted as associations between biomarkers and disease severity within the PD cohort and not as evidence of biomarker elevation or reduction relative to the general population. In addition, potential residual confounding related to renal function, dietary factors, medications, metabolic conditions, and lifestyle variables known to influence serum uric acid concentrations could not be completely excluded. The limited number of advanced disease cases may also have affected the stability of the multivariable regression estimates. Furthermore, no formal correction for multiple statistical comparisons was applied, which may have increased the risk of type I error. Another important limitation is that inflammatory biomarkers were measured in peripheral blood; therefore, peripheral cytokine expression may not directly reflect inflammatory activity within the central nervous system and should be interpreted as an indicator of systemic rather than central neuroinflammation. Consequently, the findings should be interpreted with caution and considered preliminary, warranting confirmation in larger longitudinal studies that include appropriate control populations.

## Conclusions

The present study identified significant associations between peripheral inflammatory cytokine expression, serum uric acid levels, and clinical disease severity in PD. Higher TNF-α and IFN-γ expression levels were associated with greater disease severity, whereas lower serum uric acid levels demonstrated strong inverse associations with both disease severity and inflammatory marker expression. These findings suggest a relationship between systemic inflammation, oxidative stress, and the clinical manifestations of PD. The combined assessment of TNF-α, IFN-γ, and serum uric acid represents a promising area for future biomarker research; however, the clinical utility and prognostic value of these markers remain to be established. Further validation in larger, multicenter, longitudinal studies with appropriate control populations is required. Additional research is also needed to clarify the biological mechanisms underlying these associations and to evaluate whether inflammatory and oxidative stress pathways may represent potential therapeutic targets in PD.
